# Integrative Analysis of Iso-Seq and RNA-Seq Identifies Key Genes Related to Fatty Acid Biosynthesis and High-Altitude Stress Adaptation in *Paeonia delavayi*

**DOI:** 10.3390/genes16080919

**Published:** 2025-07-30

**Authors:** Qiongji He, Wenjue Yuan, Rui Wang, Wengao Yang, Guiqing He, Jinglong Cao, Yan Li, Lei Ye, Zhaoguang Li, Zhijiang Hou

**Affiliations:** 1 Institute of Alpine Economic Plant, Yunnan Academy of Agricultural Sciences, Lijiang 674199, China; heqiongji@163.com (Q.H.); ywj112089@163.com (W.Y.);; 2 Hefu Town Comprehensive Service Center, Huzhou 313000, China; 3 Yunnan Academy of Agricultural Sciences, Kunming 650051, China

**Keywords:** *Paeonia delavayi*, Iso-seq, RNA-seq, fatty acid biosynthesis, altitude

## Abstract

**Background/Objectives:** *Paeonia delavayi*, a high-altitude-adapted medicinal and oil-producing plant, exhibits broad elevational distribution. Understanding how environmental factors regulate its growth across altitudes is critical for optimizing cultivation and exploiting its economic potential. **Methods:** In this study, we conducted a comprehensive Iso-Seq and RNA-seq analysis to elucidate the transcriptional profile across diverse altitudes and three seed developmental stages. **Results:** Using Pacbio full-length cDNA sequencing, we identified 39,267 full-length transcripts, with 80.03% (31,426) achieving successful annotation. RNA-seq analysis uncovered 11,423 and 9565 differentially expressed genes (DEGs) in response to different altitude and developmental stages, respectively. KEGG analysis indicated that pathways linked to fatty acid metabolism were notably enriched during developmental stages. In contrast, pathways associated with amino acid and protein metabolism were significantly enriched under different altitudes. Furthermore, we identified 34 DEGs related to fatty acid biosynthesis, including genes encoding pivotal enzymes like biotin carboxylase, carboxyl transferase subunit alpha, malonyl-CoA-acyl carrier protein transacylase, 3-oxoacyl-ACP reductase, 3-hydroxyacyl-ACP dehydratase, and stearoyl-ACP desaturase enoyl-ACP reductase. Additionally, ten DEGs were pinpointed as potentially involved in high-altitude stress response. **Conclusions:** These findings provide insights into the molecular mechanisms of fatty acid biosynthesis and adaptation to high-altitude stress in peony seeds, providing a theoretical foundation for future breeding programs aimed at enhancing seed quality.

## 1. Introduction

*P. delavayi*, a member of the *Moutan* DC section within the genus *Paeonia* and family Paeoniaceae [1], is a distinctive wild peony species endemic to China. It thrives in sunny mountain slopes and grasslands, with an impressive altitudinal range from 1850 to 4000 m, which is the broadest among its relatives [2]. The MaxEnt model predicts that *P. delavayi*’s optimal distribution is primarily in Yunnan, Sichuan, Guizhou, and the Tibet Autonomous Region of China, with a notable concentration in specific areas of Yunnan province [3,4]. Characterized by its large, vibrant flowers that bloom in a spectrum of colors from white to deep red, *P. delavayi* is prized both for its ornamental value and for the rich oil content found in its seeds. In 2023, He et al. [5] bred a new cultivar, ‘Lidan 1’, derived from a wild *P. delavayi* plant, which boasts an oil content of up to 20.7% and is well-suited for cultivation between altitudes of 2300 and 3400 m. Its genetic diversity and adaptability make it a valuable resource for botanical research and conservation efforts.

Peony seed oil has been recognized as a new functional food resource since 2011 and became an edible vegetable oil for its rich content of unsaturated fatty acids, especially α-linolenic acid (ALA) which can lower blood sugar and lipids, aid weight loss, prevent cardiovascular diseases, and combat cancer [6,7,8,9]. Moreover, its potential as a biofuel source underscores its importance in the renewable energy sector. Therefore, tree peony is an important economic plant with ornamental, medical, and oil applications [10]. The main oil tree peony cultivars promoted in China are *Paeonia ostii* ‘Fengdan’ and *Paeonia rockii* [11,12]. As a close relative of *P. ostii* ‘Fengdan’, *P. delavayi* also possesses significant potential for oil production. The content of unsaturated fatty acids in *P. delavayi* seed oil was 89.34%, comprising 14.25% linoleic acid and 72.26% linolenic acid, higher than that in *P. ostii* ‘Fengdan’ seed oil [13]. The cultivation and utilization of *P. delavayi* seed oil not only contributes to economic development, but also promotes sustainable agricultural practices and biodiversity conservation.

Plant phenotypic diversity is shaped by the interplay of genetic and environmental factors. Previous studies on *Paeonia ludlowii* [14] and *Paeonia szechuanica* [15] have highlighted variations in oil content of peony seeds from different geographic locations. Understanding the environmental regulatory mechanisms is, therefore, pivotal for deciphering fatty acid synthesis pathways. Indeed, recent RNA-seq studies have shed light on key genes in peony oil synthesis pathways. For instance, Li et al. identified 388 genes potentially involved in de novo fatty acid and triacylglycerol biosynthesis [16]. Notably, three genes (*SAD*, *FAD2*, and *FAD8*) encoding fatty acid desaturases exhibited high expression levels during the rapid oil accumulation stage compared to the initial stage of seed development. Furthermore, the overexpression of *PrFAD2* and *PrFAD3* in *Arabidopsis* has been shown to enhance linoleic and α-linolenic content [17,18]. Mei et al. retrieved three *PoKAS* genes from transcriptome data and confirmed their significant role in peony seed fatty acid synthesis by using qRT-PCR analysis [19].

The advancement of sequencing technologies has established RNA-seq as a cornerstone in the study of gene expression regulation [20]. However, due to the limitations of short reads, the method’s reliance on short reads can sometimes lead to assembly errors, including misidentified splice sites and the formation of chimeric transcripts, which may result in incomplete transcriptome coverage [21]. To address these challenges, we employed PacBio Iso-Seq technology, which simplifies the analysis of non-reference transcriptomes and effectively circumvents the need for a reference genome [22]. In this study, *P. delavayi* seeds were collected at three distinct developmental stages across varying altitudes. Both RNA-seq and PacBio Iso-Seq were utilized to identify differentially expressed genes (DEGs) that are implicated in the lipid metabolism and in response to high-altitude stress. Our comprehensive approach unveiled the transcriptomic landscape of fatty acid biosynthesis in peony seeds and identified potential candidate genes that play a role in adapting to high-altitude conditions. These insights are not only pivotal for guiding oil selection in peony cultivation, but also serve as a theoretical foundation for enhancing the quality of future breeding programs.

## 2. Materials and Methods

### 2.1. Plant Materials and Measurement of Traits

Three healthy and well-growing *P. delavayi* plants were randomly selected from each of the two altitudes (2400 m and 3400 m). At seed maturity, 13 yield-related trait indicators were measured and recorded for each plant, including plant height, number of fruiting branches per plant, number of pods per plant, number of carpels per pod, number of seeds per carpel, number of seeds per pod, seed-setting rate, abortion rate, seed length, seed width, seed thickness, single-seed mass, and single-plant seed mass. For each plant, after counting the number of pods and carpels, the carpels were dissected to count the seeds in each carpel. Seed development was observed, with well-developed and expanded seeds recorded as viable seeds, while underdeveloped or non-expanded seeds were recorded as aborted seeds. The seed-setting rate and abortion rate were then calculated based on these observations.

**Figure 1:**
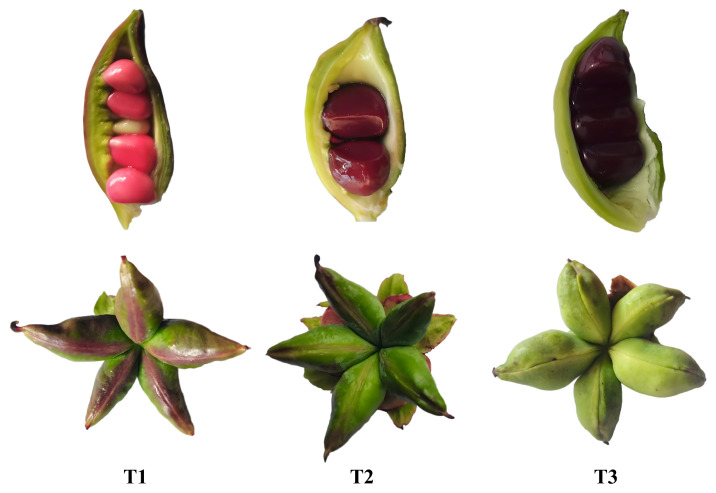
Morphological characterization of *P. delavayi* seeds at three developmental stages.

Meanwhile, three *P. delavayi* plants at different developmental stages were sampled from altitudes of 2400 m and 3400 m, respectively, labeled as M1, M3, and M5 and M2, M4, and M6. Each sampling occurred one month apart. Three independent *P. delavayi* plants were sampled for each developmental stage and altitude, with three biological replicates per plant (total *n* = 18) used for RNA-seq. Mixed samples from the same plant were subsequently used for Iso-seq. All seed samples were frozen in liquid nitrogen and stored at −80 °C until RNA extraction. The three developmental stages were identified based on key morphological characteristics: the yellow maturation stage (characterized by a pale yellow to pink variegated pericarp coloration with partial translucency), the physiological maturity stage (marked by a fully hardened, uniform brown seed coat), and the post-abscission stage (exhibiting complete blackening of the seed coat with pronounced desiccation features) (Figure 1).

### 2.2. RNA Extraction and Assessment

High-quality RNA is crucial for successful sequencing. Total RNA was extracted from seed samples using Trizol following the manufacturer’s instructions. The integrity of the extracted RNA was assessed by 1% agarose gel electrophoresis, and the concentration and purity were assessed using Nanodrop 2000 and Agilent 2100. Only RNA samples with an optical density (OD) 260/280 ratio between 1.8 and 2.2, RNA Integrity Number (RIN) ≥ 8, and RNA yield > 1 μg, and concentration of ≥200 ng/μL were used for RNA-Seq library construction. For PacBio Iso-Seq, total RNA was isolated from 18 samples and mixed in equal amounts, respectively.

### 2.3. PacBio Iso-Seq Library Construction, Sequencing, and Annotation

The PacBio Iso-Seq library was constructed according to the official protocol, with specific adjustments made as follows: The mixed RNA sample was reverse-transcribed from mRNA to synthesize full-length cDNA using the Clonetech SMARTer™ PCR cDNA Synthesis Kit. Primers containing Oligo dT were used for A-T base pairing with the polyA tail, serving as the primer for the reverse transcription of cDNA. Following purification with PB beads, the cDNA products shorter than 1 kb length were eliminated. The cDNA was purified and repaired at its ends, followed by the addition of SMRT adapters and ligation of the fragment.

The libraries were sequenced on an PacBio Sequel II platform using the SMRT (Single Molecule Real-Time) sequencing method. Raw data were processed using SMRTlink 10.1, following the Iso-Seq protocol, to obtain full-length transcript sequences. The circular consensus sequence (CCS) was generated from the subreads, and the full-length non-chimeric reads (FLNC) were used for further analysis. These FLNC reads were clustered to obtain consensus isoforms using the ICE tool of the SMRT link, and then were polished using the Arrow algorithm. Additionally, these high-quality isoforms were subjected to correction using LoRDEC 0.9 [23]. Then, CD-HIT 4.8.1 [24] was performed to eliminate redundancy. Finally, the obtained high-quality full-length transcripts were annotated using Diamond 0.9.04 [25] against public databases with an E-value threshold of 1 × 10^−5^. These databases included NCBI non-redundant protein sequences (NR), Swiss-Prot, EuKaryotic Orthologous Groups (KOG), Gene Ontology (GO), and the Kyoto Encyclopedia of Genes and Genomes (KEGG).

### 2.4. RNA-Seq Analysis

The cDNA libraries were constructed using high-quality RNA and were sequenced on the BGI DNBSEQ-T7 platform. For the RNA-seq data analysis, raw reads were preprocessed using SOAPnuke 2.1.0 [26]. Clean reads were aligned to the polished transcript sequences derived from PacBio ISO-seq using Bowtie2 [27]. The expression levels of isoforms were calculated with RSEM [28], and read counts were normalized to FPKM (Fragments Per Kilobase per Million bases) values.

Differential expression analysis was performed using the DESeq2 package [29] with |log2 (Fold Change)| > 1 and an adjusted *p* value < 0.05. In additional, these identified differentially expressed genes (DEGs) were subsequently subjected to GO and KEGG enrichment analysis using Goatools [30] and KOBAS (http://bioinfo.org/kobas/%EF%BC%89, accessed on 3 July 2025), respectively. Benjamini–Hochberg adjusted *p*-values (*q*-value) below 0.05 were used to indicated significance. Conversely, the *p*-value was referenced when no significantly enriched terms or pathways were identified using the *q*-value.

### 2.5. Gene Expression Validation Using qPCR

To assess the reliability of the RNA-seq data, 12 differentially expressed genes associated with fatty acid regulation were selected for validation via qRT-PCR. Specific primers for each of the 12 selected genes were designed using Primer 5.0 software (Table S1). The reaction mixture, with a final volume of 20 µL, included 10 μL of TB Green Premix Ex Taq II, 1.0 μL of cDNA, 1.0 μL of each primer (at a concentration of 10 μM), and 6.0 μL of sterilized deionized water. The PCR cycle program was set to an initial denaturation at 94 °C for 5 min, followed by 35 cycles of denaturation at 94 °C for 30 s, annealing at 60 °C for 30 s, and extension at 72 °C for 30 s, concluding with a final elongation at 72 °C for 5 min. The analysis of gene expression was performed using the 2^−ΔΔCT^ method.

## 3. Results

### 3.1. The Difference in Traits Between Different Altitudes

Through observations of fruit and seed traits at maturity for *P. delavayi* plants at two elevations (2400 m and 3400 m) (Table 1), we found that all 13 yield-related traits of fruits and seeds at 3400 m were significantly superior to those at 2400 m. Specifically, the number of fruiting branches per plant, number of seeds per pod, seed length, single-seed mass, and single-plant seed mass at 3400 m were markedly higher than those at 2400 m (*p* < 0.05).

### 3.2. PacBio ISO-Seq Analysis

**Figure 2:**
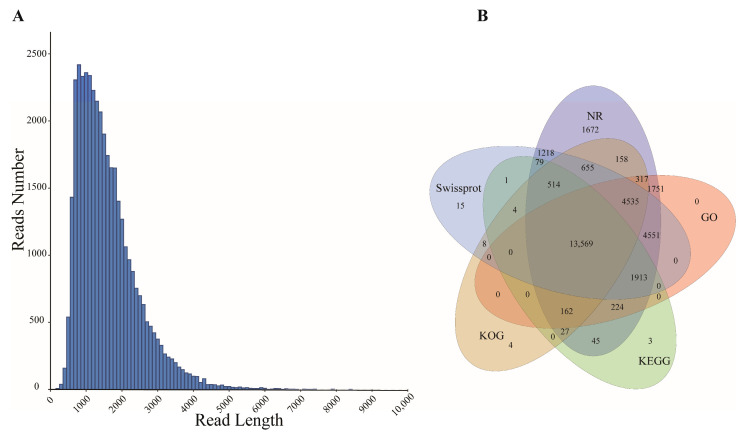
(**A**) Length distribution of PacBio Iso-Seq-assembled transcripts. (**B**) Venn plot of isoform numbers annotated to five databases (NR, Swiss-Prot, KOG, GO, KEGG).

PacBio ISO-seq sequencing of six *P. delavayi* seeds was performed. In total, 39.78–63.82 Gb of raw data were generated for each sample, a total of 171,594,853 subreads were generated, and then 3,038,301 CCS reads were obtained. Finally, 186,254 sequences were identified as FLNC sequences (Table 2). The results from each library were merged. The isoforms were corrected using LoRDEC, and redundancy was removed using CD-HIT. Finally, the full-length transcripts contained 39,267 isoforms, with an average length of 1550 bp, which were considered as the reference transcripts and were used for further analysis (Table S2). The N50 value of these transcripts was 1808 bp, exceeding the average length (Figure 2A).

### 3.3. Function Annotation of Full-Length Transcripts

The Diamond blastx tool was utilized to align isoforms against the NR, GO, KEGG, KOG, and the Swiss-Prot protein databases for functional annotation. Among the 39,267 isoforms, 31,426 (80.03%) were annotated in at least one database, with 31,391 (79.94%) annotated to NR, 27,022 (68.81%) to GO, 16,541 (42.12%) to KEGG, 19,954 (50.81%) to KOG, and 27,063 (68.92%) to Swiss-Prot, and 13,569 isoforms were annotated across all databases (Figure 2B). The results of NR annotation show the highest homology between *P. delavayi* and *Vitis vinifera* (Figure S1A).

Among the KOG annotations, 19,954 isoforms were distributed across 26 KOG categories. The most abundant term was “general function prediction only”, encompassing 3502 isoforms (Figure S1B). In the GO analysis, a total of 27,022 isoforms were assigned to 41 GO terms, including 25 terms to the biological process, 2 terms to the cellular component, and 14 terms to the molecular function. The top three largest GO terms were cellular anatomical entity (19,242), cellular processes (14,181) and binding (13,589) (Figure S1C). In the KEGG classification, 16,541 isoforms were classified into 18 level-2 KEGG pathways. The top five were carbohydrate metabolism (1780), translation (1521), folding, sorting, and degradation (1516), amino acid metabolism (965), and transport and catabolism (787). Notably, 749 isoforms were mapped to the lipid metabolism pathway, providing valuable insights into fatty acid regulation in *P. delavayi* (Figure S1D).

### 3.4. RNA-Seq Analysis of P. delavayi Seeds

*P. delavayi* seeds at three different developmental stages were collected at two different altitudes (2400 m, 3400 m). Each group had three biological replicates. The transcriptome of 18 samples was sequenced on the BGI DNBSEQ-T7 platform, generating 1,339,334,262 raw reads. After filtering out low-quality reads, a total of 1,099,046,344 clean paired-end reads were obtained. The lowest Q20 percentage, Q30 percentage, and GC percentage were 98.77%, 95.95%, and 45.05%, respectively (Table S3). The clean reads were mapped to the full-length transcripts, 83.84–89.76% were mapped to the reference transcripts, and 13.59–26.88% were uniquely mapped reads (Table S4).

### 3.5. Identification of DEGs

**Figure 3:**
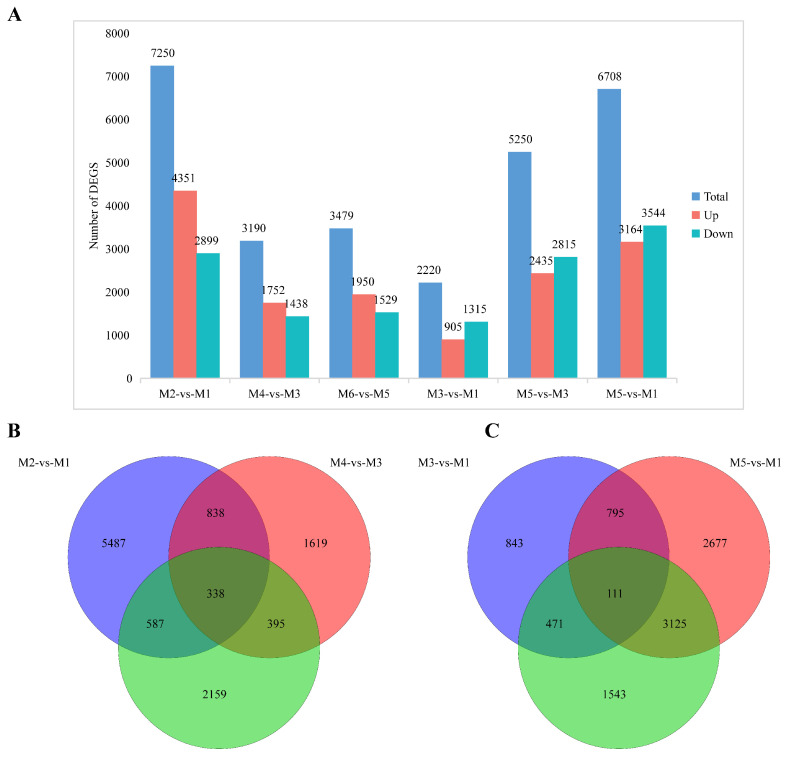
(**A**) Number of DEGs in pairwise comparisons across altitudes (M2 vs. M1, M4 vs. M3, M6 vs. M5). (**B**) Venn plot of DEGs at different altitude comparisons (M2 vs. M1, M4 vs. M3, and M6 vs. M5). (**C**) Venn plot of DEGs in different developmental stage comparisons (M3 vs. M1, M5 vs. M3, and M5 vs. M1).

Differential expression analysis was initially performed between different altitudes, i.e., M2 vs. M1, M4 vs. M3, and M6 vs. M5 comparisons. The results show that 7250 DEGs (4351 up-regulated and 2899 down-regulated) were identified in the M2 vs. M1 comparison, 3190 DEGs (1752 up-regulated and 1438 down-regulated) were identified in the M4 vs. M3 comparison, and 3479 DEGs (1950 up-regulated and 1529 down-regulated) were identified in the M6 vs. M5 comparison (Figure 3A). In all three comparison groups, the number of up-regulated genes exceeded that of down-regulated genes, and the number of DEGs was notably higher in the seed development stage than in the seed maturation stage. A Venn diagram revealed a total of 11,423 DEGs across the three comparisons, with 5487 unique to M2 vs. M1, 1619 unique to M4 vs. M3, 2159 unique to M6 vs. M5, and 338 DEGs differing significantly across all three comparisons (Figure 3B).

To investigate the DEGs in seeds at different developmental stages at the same altitude, differential expression analyses in M3 vs. M1, M5 vs. M3, and M5 vs. M1 comparisons were performed. A total of 2220 DEGs were found in the M3 vs. M1 comparison, with 905 up-regulated and 1315 down-regulated. In total, 5250 DEGs, with 2435 up-regulated and 2815 down-regulated, were identified in the M5 vs. M3 comparison, and 6708 DEGs, with 3164 up-regulated and 3544 down-regulated, were identified in the M5 vs. M1 comparison. Overall, 9565 DEGs were found across the three comparisons, with 843 unique DEGs unique in M3 vs. M1, 1543 unique DEGs in M5 vs. M3, and 2677 unique DEGs in M5 vs. M1, and 111 DEGs were identified across all three comparisons (Figure 3C).

### 3.6. Enrichment Analysis of DEGs

To explore the biological processes involved in seed development and high-altitude adaptation, KEGG enrichment analysis was performed on these identified DEGs. In comparisons between different altitudes, a total of 126 KEGG pathways were enriched in the M2 vs. M1 comparison, with only four showing significant enrichment (*q* value ≤ 0.05): arginine biosynthesis; nitrogen metabolism; alanine, aspartate, and glutamate metabolism; and protein processing in endoplasmic reticulum. In the M4 vs. M3 comparison, a total of 112 KEGG pathways were enriched, with two showing significant enrichment: glycolysis/gluconeogenesis and protein processing in endoplasmic reticulum. In the M6 vs. M5 comparison, a total of 121 KEGG pathways were enriched, with two showing significant enrichment: isoflavonoid biosynthesis and MAPK signaling pathway—plant (Figure S2A, Table S5).

KEGG enrichment analysis in comparisons between different developmental stages at an altitude of 2400 m was performed, and the results show that the 7, 12, and 21 pathways were involved in secondary metabolites biosynthesis, signal transduction, and defense-related response, as well as showing that the fatty acid, amino acid, and saccharide metabolisms were significantly enriched in the M3 vs. M1, M5 vs. M3, and M5 vs. M1 comparisons, respectively. Detailed information is listed in Table S6 and Figure S2. Remarkably, monoterpenoid biosynthesis and pathways related to fatty acid metabolism were found to be enriched across all three comparisons. Specifically, fatty acid degradation and alpha-linolenic acid metabolism were significantly enriched in the M3 vs. M1 comparison, and fatty acid biosynthesis was significantly enriched in the M5 vs. M3 and M5 vs. M1 comparisons.

### 3.7. Analysis of DEGs at Varying Altitudes and Different Developmental Stages of Seeds

**Figure 4:**
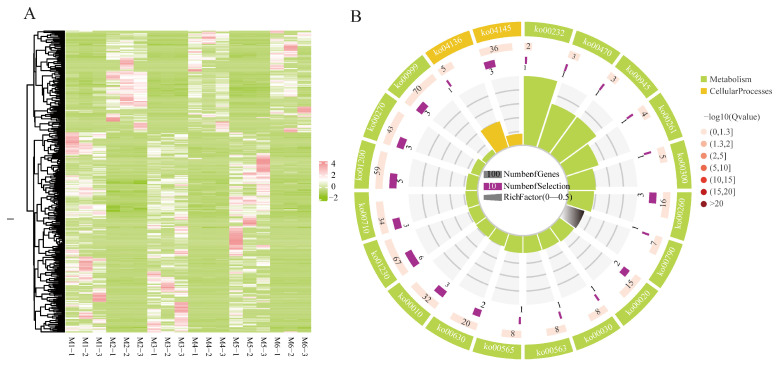
(**A**) Expression heatmap of DEGs identified among comparisons at different altitudes (pink: high expression; green: downregulation). (**B**) KEGG enrichment analyses on DEGs identified among the comparisons at different altitudes.

In the comparisons at different altitudes, 338 genes were significantly expressed across all three comparisons. Clustering analysis indicated a similar transcriptional pattern at the same altitude (Figure 4A). In KEGG enrichment, they were mapped into 17 KEGG pathways, mainly categorized within global and overview maps and carbohydrate metabolism, and six pathways were significantly enriched with *p* values ≤ 0.05, including biosynthesis of amino acids, glycine, serine, and threonine metabolism, carbon metabolism, glycolysis/gluconeogenesis, caffeine metabolism, and carbon fixation in photosynthetic organisms (Figure 4B). Furthermore, 10 out of 338 DEGs were found to be related to these six pathways. Among them, six DEGs showed up-regulated expression at 2400 m altitude, whereas four involved in carbon metabolism, carbon fixation in photosynthetic organisms, biosynthesis of amino acids, and caffeine metabolism were down-regulated at 2400 m. Detailed information of these ten DEGs is listed in Table S7.

**Figure 5:**
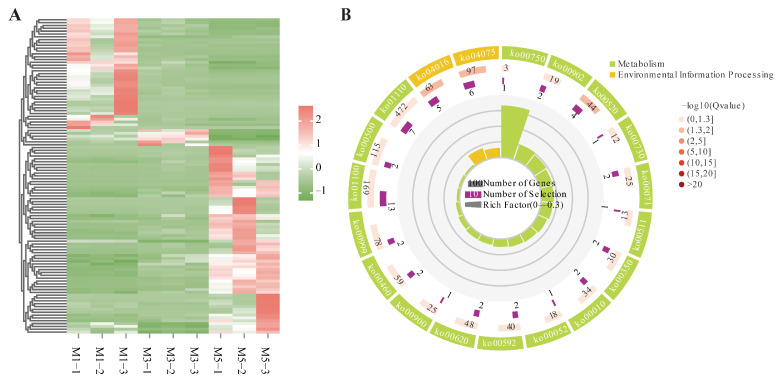
(**A**) Expression heatmap of 111 DEGs identified among the comparisons at different developmental stages. (**B**) KEGG enrichment analyses on DEGs identified among the comparisons at different developmental stages.

In the comparisons of different developmental stages of seeds, 111 DEGs were obtained from the intersection analysis of the three comparisons. In clustering analysis, almost the opposite expression patterns were observed in the M1 and M5 groups, and only six genes showed up-regulated expression in M3 (Figure 5A). These 111 DEGs were enriched into pathways mainly categorized within global and overview maps and signal transduction. Among them, five pathways were significantly enriched with *p* values ≤ 0.05, including the MAPK signaling pathway: plant, plant hormone signal transduction, amino sugar and nucleotide sugar metabolism, monoterpenoid biosynthesis, and vitamin B6 metabolism (Figure 5A). A total of thirteen DEGs were identified within these pathways, three of which exhibited increasing expression as the seed developed, while the other ten genes exhibited the opposite pattern. Detailed information of these 13 DEGs is listed in Table S8.

### 3.8. Key Genes Associated with Fatty Acid Biosynthesis

In the transcriptome analysis of seeds at different developmental stages, thirteen KEGG pathways related to lipid metabolism were identified (Table S9), among which only three pathways were significantly enriched: fatty acid degradation (M3 vs. M1), alpha-linolenic acid metabolism (M3 vs. M1), and fatty acid biosynthesis (M5 vs. M3 and M5 vs. M1). It was found that the number of DEGs in the three pathways mentioned above increased with seed development, and the number of up-regulated genes exceeded that of down-regulated genes in the three comparisons. Although fatty acid biosynthesis was not significantly enriched in the M3 vs. M1 comparison group, it still contained a large number of DEGs, suggesting potential for subsequent key candidate gene screening.

**Figure 6:**
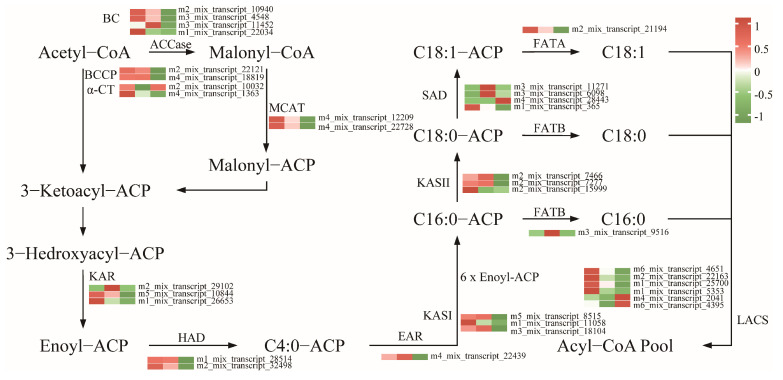
Proposed gene networks involved in fatty acid biosynthesis in *P. delavayi*. The expression levels (represented by the FPKM) of the possible candidates are highlighted by color scales in *P. delavayi* seeds at different development stages (M1, M3, and M5). BC: biotin carboxylase; BCCP: biotin carboxyl carrier protein; α-CT: carboxyl transferase subunit alpha; MACT: malonyl-CoA-acyl carrier protein transacylase; KAR: 3-oxoacyl-ACP reductase; HAD: 3-hydroxyacyl-ACP dehydratase; EAR: enoyl-ACP reductase; KASII: 3-oxoacyl-ACP synthase II; FATB: fatty acyl-ACP thioesterase B; FATA: fatty acyl-ACP thioesterase A; SAD: stearoyl-ACP desaturase; LACS: long-chain acyl-CoA synthetase.

To further uncover the key genes associated with the biosynthesis of fatty acids, the DEGs involved in fatty acid biosynthesis pathway were screened and clustered, yielding 34 genes, including eight DEGs encoding ACCase subunits: four biotin carboxylase (*BC*), two carboxyl transferase subunit alpha (*α-CT*), and two biotin carboxyl carrier protein (*BCCP*); two DEGs encoding malonyl-CoA: ACP transacylase (*MACT*); three DEGs encoding 3-oxoacyl-ACP synthase I (*KASI*); three DEGs encoding 3-oxoacyl-ACP synthase II (*KASII*); three DEGs encoding 3-oxoacyl-ACP reductase (*KRA*); two DEGs encoding 3-hydroxyacyl-ACP dehydratase (*HAD*); one DEG encoding enoyl-ACP reductases (*EAR*); six DEGs encoding long-chain acyl-CoA synthetase (*LACS*); four DEGs encoding stearoyl-ACP desaturase (*SAD*); and two DEGs encoding other components of FA synthase (Table S10). These encoded key enzymes in fatty acid biosynthesis are shown in Figure 6. Most of these genes were highly expressed in the early and middle stages of seed development and decreased in the later stages of seed development. And two genes encoding *SAD* (m3_mix_transcript_11271 and m3_mix_transcript_6098) exhibited a pattern of initial increase followed by a subsequent decrease as seed development progressed, displaying a bell-shaped curve with seed development over time.

### 3.9. Validation of RNA-Seq by qRT-PCR

**Figure 7:**
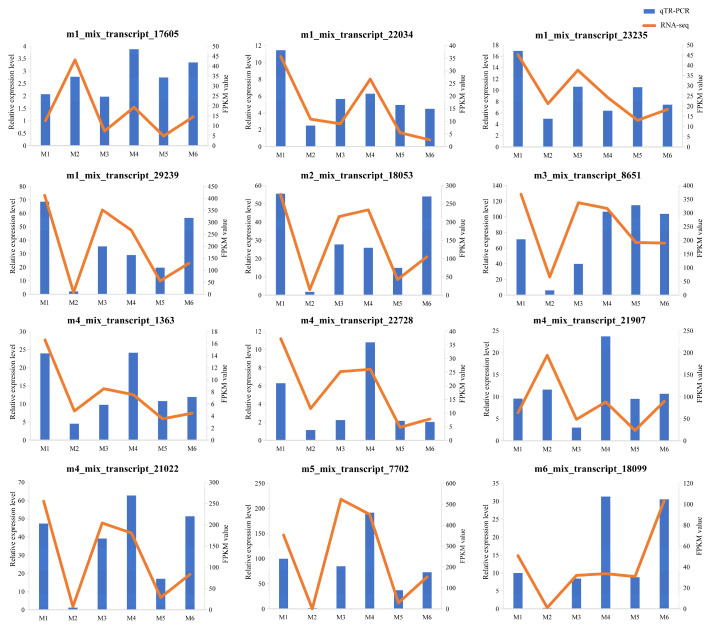
The expression levels of 12 DEGs in qRT-PCR and RNA-seq. The column chart shows the relative expression levels of qRT-PCR (left *y*-axis). The broken line graph shows the expression levels of RNA-Seq (right *y*-axis). Data represent means of three replicates.

To verify the reliability of the RNA-Seq data, 12 DEGs were subjected to qRT-PCR analysis. The gene expression trends detected by RNA-Seq and qRT-PCR were consistent, providing support for the credibility and accuracy of the RNA-Seq results (Figure 7).

## 4. Discussion

Understanding the pivotal stages of seed development and the underlying molecular mechanisms of oil synthesis is essential for future enhancements in the yield and quality of tree peony oil [31]. Previous research has documented significant variations in fatty acid profiles as seeds mature [32]. Peony seeds, akin to other oil-bearing plants, experience three distinct phases of lipid accumulation: an early developmental stage with low fatty acid levels, a mid-stage characterized by a significant increase in oil accumulation, and a final stage of natural desiccation where the oil content stabilizes [16]. The seed’s physical characteristics and chemical compound compositions undergo substantial changes during different growth stages, accompanied by fluctuations in gene expression related to fatty acid metabolism [33]. Wang et al. [31] and Meng et al. [34] identified five primary fatty acids in tree peony seeds: palmitic acid (C16:0, PA), stearic acid (C18:0, SA), oleic acid (C18:1, OA), linoleic acid (C18:2, LA), and α-linolenic acid (C18:3, ALA). The concentrations of these fatty acids initially increase and then decrease, peaking during the mid-stage of seed maturation.

In general, lipid biosynthesis in oil seed plants primarily involves three stages: de novo fatty acid synthesis, triacylglycerol (TAG) assembly, and oil body formation [35]. Over 30 enzymatic reactions are required to produce C16 or C18 fatty acids from acetyl-CoA and malonyl-CoA, involving key enzymes such as ACCase (including *BC*, *BCCP*, *α-CT* and *β-CT*), *MCAT*, *KAS* (including *KASI*, *KASII*, and *KASIII*), *KAR*, *HAD*, *EAR*, and *FAT* (including *FATA* and *FATB*) [36]. *KAS*, a key enzyme in the fatty acid synthesis pathway, is crucial for lipid accumulation [19]. *KASI* enzymes facilitate six additional condensation reactions, resulting in the production of C16:0-ACP and *KASII* enzymes catalyze the conversion of C16:0-ACP into C18:0-ACP [37]. In this study, the key genes responsible for encoding the above enzymes were pinpointed during the seed development (Figure 6). The expression of those genes was high during the early and middle stages and significantly decreased in later stages, including those encoding *BC*, *BCCP*, *α-CT*, *MACT*, *KASI*, *KASII*, *HAD*, and *EAR*, indicating that fatty acid accumulation in peony seeds mainly occurs during the early and middle stages. Additionally, two transcripts encoding stearoyl-acyl carrier protein desaturases (SADs) exhibited a bell-shaped expression pattern during seed development, with low expression in the early and late stages and up-regulated expression in the middle stages. *SAD* regulates the initial desaturation step from saturated to unsaturated fatty acids, playing a key role in alpha-linolenic acid biosynthesis [38]. The activity of *SAD* increased during the middle phase of seed growth, potentially facilitating the conversion of C18:0-ACP to C18:1-ACP. In conclusion, this study further demonstrates that the rapid synthesis and accumulation of fatty acids during the middle stage of seed development is likely due to the activation of the fatty acid synthesis pathway and the increased expression of key genes.

Altitude gradients significantly influence various environmental factors, such as cold temperatures, intense ultraviolet radiation [39], hypoxia, nutrient-poor soils, and reduced CO_2_ levels [40]. Alpine plant species often employ diverse adaptive mechanisms to counteract the stress induced by altitude variations [41]. It has been reported that protein metabolism is a critical biological process for plants to cope with environmental stress [42], which was also observed in our study, where several KEGG pathways related to protein or amino acid metabolism were significantly enriched, such as arginine biosynthesis, alanine, aspartate, and glutamate metabolism, and protein processing in endoplasmic reticulum. Moreover, high-altitude plants require additional energy to withstand environmental stress, and carbohydrate metabolism is the primary source of cellular energy [43]. PacBio Iso-Seq technology enabled precise characterization of these metabolic adaptations by generating 39,267 full-length transcripts, which resolved complete gene structures for key enzymes. We found that six pathways related to carbohydrate metabolism were significantly enriched in KEGG enrichment analysis on 111 DEGs shared by three comparisons between two altitudes, indicating that carbohydrate metabolism might play an important role in peony’s response to high altitude stress. Meanwhile, two down-regulated genes were significantly enriched in carbon metabolism and carbon fixation in photosynthetic organisms’ pathways, and one DEG (m6_mix_transcript_28189) was identified as encoding malate dehydrogenase (MDH), suggesting the involvement of MDH in the regulation of energy metabolism. MDH, an oxidation-reduction enzyme, catalyzes the reversible conversion of oxaloacetic acid to malic acid, playing crucial roles in plant development, photosynthesis, and responses to abiotic stress. MDH is believed to play a crucial role in the response of *Arachis hypogaea* to manganese toxicity stress [44]. Furthermore, under low-phosphorus stress, MDH was found to be up-regulated, contributing to fir trees’ stress response [45]. Overexpression of the *MDH* gene has also been demonstrated to enhance salt stress resistance in soybean [46]. In this study, the expression of the *MDH* gene (m6_mix_transcript_28189) was found to be low in seeds at low altitudes (M1/3/5), but significantly increased at high altitudes (M2/4/6). This observation suggests a potential positive impact of MDH on the response of *P. delavayi* to high-altitude stresses. However, the specific molecular mechanisms underlying this phenomenon remain unclear and warrant further exploration.

The study focused on two altitudes (2400 m and 3400 m) representing *P. delavayi*’s core distribution and cultivation range, where contrasting environmental stresses enable robust adaptation analysis. While this design effectively identified key adaptive genes (e.g., *MDH*, *SAD*) and pathways, future work should expand to the species’ full altitudinal range (1850–4000 m) to distinguish elevation-specific from generalized responses.

## 5. Conclusions

This study explores the transcriptomic landscape of *P. delavayi* seeds across two distinct elevations (i.e., 2400 m and 3400 m) and various developmental stages (i.e., early, middle, and late). We identified 11,423 differentially expressed genes (DEGs) when comparing different altitudes and 9565 DEGs across various developmental stages. Among these, we pinpointed 34 key genes that are integral to the biosynthesis of fatty acids, with some being annotated as encoding enzymes such as *BC*, *BCCP*, *α-CT*, *MACT*, *KASI*, *KASII*, *KRA*, *HAD*, *EAR*, and *SAD*. Furthermore, we identified ten candidate genes that may play a role in the plant’s adaptation to high-altitude stress, with four of these exhibiting notably increased expression at higher altitudes. Collectively, the insights gleaned from this study are instrumental for deciphering the critical phases of oil accumulation and the regulatory mechanisms behind fatty acid synthesis, which are pivotal for enhancing the yield and quality of tree peony oil.

## Figures and Tables

**Table 1:** Fruit and seed traits at maturity of *P. delavayi* at different altitudes.

Altitude	2400 m	3400 m
Height (cm)	121.00 ± 5.30 a	133.67 ± 12.66 a
No. of bearing branches	6.00 ± 1.00 b	9.67 ± 1.15 a
No. of pods	9.67 ± 0.58 a	10.33 ± 0.58 a
No. of carpels	4.33 ± 1.53 a	5.00 ± 1.00 a
No. of seeds	7.00 ± 1.00 a	7.33 ± 1.53 a
No. of seeds per pod	30.00 ± 1.00 b	35.00 ± 1.00 a
Setting rate/%	67.72 ± 2.87 a	72.45 ± 3.62 a
Abortion rate/%	32.28 ± 2.87 a	27.55 ± 3.62 a
Seed length (cm)	1.33 ± 0.036 b	1.48 ± 0.09 a
Seed width (cm)	1.22 ± 0.04 a	1.26 ± 0.02 a
Seed thickness(cm)	1.06 ± 0.04 a	1.15 ± 0.05 a
Weight of per seed (g)	1.02 ± 0.05 b	1.37 ± 0.04 a
Weight of seeds (g)	215.00 ± 4.58 b	346.67 ± 6.66 a

Note: Values with the same letter are not significantly different at *p* < 0.05 level.

**Table 2:** Overview of the Pacbio Iso-seq data.

Library	Total Bases (Gbp)	Subreads Number	CCS Reads Number	Full-Length Non-Chimeric Reads
m1_mix	46.28	27,846,502	522,779	33,805
m2_mix	63.82	36,685,495	624,399	42,187
m3_mix	41.75	26,623,625	463,744	25,347
m4_mix	39.78	24,704,351	451,654	28,679
m5_mix	38.17	24,116,939	433,280	24,677
m6_mix	55.83	31,617,941	542,445	31,559
Total	285.63	171,594,853	3,038,301	186,254

## Data Availability

The raw data generated by RNA-seq in this study is available in the NCBI database with the accession number under PRJNA1147466.

## Supplementary Materials

The following supporting information can be downloaded at: https://www.mdpi.com/article/10.3390/genes16080919/s1. Figure S1: Functional annotation of full-length transcripts in *P. delavayi*. (A) Species distribution of NR annotation; (B) KOG functional classification; (C) GO term enrichment; (D) KEGG pathway classification. Figure S2: KEGG Pathway Enrichment Analysis of DEGs Under Different Altitudes (M2 vs. M1, M4 vs. M3, M6 vs. M5) and Seed Developmental Stages (M3 vs. M1, M5 vs. M3, M5 vs. M1). Table S1: Sequences of primers used in the qRT-PCR analysis. Table S2: Overview of the isoforms obtained from the pooled sequencing. Table S3: RNA-seq data quality summary table. Table S4: Information of RNA-seq reads mapped with the reference transcriptome. Table S5: The significantly enriched pathways across different altitudes. Table S6: The significantly enriched pathways at different developmental stages of seeds. Table S7: The DEGs in significantly enriched pathways at different altitude. Table S8: The DEGs in significantly enriched pathways at different developmental stages of seeds. Table S9: Statistical chart of lipid metabolism pathway. Table S10: Key genes associated with fatty acid biosynthesis.
