# Supplementary material for: Integrative Analysis of Iso-Seq and RNA-Seq Identifies Key Genes Related to Fatty Acid Biosynthesis and High-Altitude Stress Adaptation in Paeonia delavayi
Source: Genes (Basel). 2025 Jul 30;16(8):919. doi: 10.3390/genes16080919 (PMC12385284; doi:10.3390/genes16080919)
Supplement: Supplementary file 1 [file genes-16-00919-s001.zip › Supplementary files/Supplementary Table.pdf]

Supplementary Table S1. Sequences of primers used in the qRT-PCR analysis

Isoforms	Description	Primer ID	Primer sequences (5′− 3′)
m1_mix_transcript_17605	very-long-chain enoyl-CoA reductase isoform X2	F	GGGAATGGAGGGTACCAAATC
		R	TTGAATCCCACCCACTGATAAA
m1_mix_transcript_22034	acetyl-CoA carboxylase BC subunit	F	CGACAACGATCGAGTACCATAAA
		R	CCTCCTCGTGCTTTGGAATAA
m1_mix_transcript_23235	cycloartenol-C-24-methyltransferase	F	TTGAGGTCATGTGGGAGAAAG
		R	CCAAGAGCTGTAAGTCGGAAA
m1_mix_transcript_29239	fatty acid desaturase 2	F	AGTAGGTGCTTTCCGTGTTTAG
		R	CGAACTAACACTCCGCGATAA
m2_mix_transcript_18053	fatty acid desaturase 2	F	ATGGAAGGAAGTAGGTGCTTTG
		R	CGAACTAACACTCCGCGATAAG
m3_mix_transcript_8651	stearoyl-ACP desaturase	F	GGAGACATGGTCACAGAAGAAG
		R	TCCAAACTGCCCAAGAAGTAA
m4_mix_transcript_1363	acetyl-coenzyme A carboxylase carboxyl transferase subunit alpha, chloroplastic	F	CCTGGACCTCACAACAGATAAA
		R	TAGCATTCGATGCCGAAGTAG
m4_mix_transcript_22728	malonyl-CoA-acyl carrier protein transacylase, mitochondrial isoform X2	F	CGTGAGGGAGGACAACAAATA
		R	GCTGAAAGCTCCAGCAAATG
m4_mix_transcript_21907	Glycolipid transfer protein domain	F	CTGCCGCTTTGGAAGAAATG
		R	GAGTCTGCAGGTATCCAAGAAA
m4_mix_transcript_21022	fatty acid desaturase 2	F	AGTAGGTGCTTTCCGTGTTTAG
		R	CGAACTAACACTCCGCGATAA
m5_mix_transcript_7702	omega-3 fatty acid desaturase	F	TAGAGGAGGGCTTACGACAA
		R	GATTTGAGGGAAGAGGTGATGT
m6_mix_transcript_18099	omega-3 fatty acid desaturase	F	TGACTTGCCCTGGTTGAATAG
		R	CTGATGGTGAGTTCTGTGACTT

Supplementary Table S2. Overview of the isoforms obtained from the pooled sequencing.

Total number	Total bases (bp)	Maximum length (bp)	Minimum length (bp)	Average length (bp)	N50 length (bp)
39,267	60,848,470	8,390	136	1,550	1,808

Supplementary Table S3. RNA-seq data quality summary table

Sample	Raw reads	Clean reads	Effective rate(%)	Q20(%)	Q30(%)	GC Content(%)
M1-1	72,957,888	64,285,618	88.11	98.8	96.07	46.37
M1-2	86,090,656	66,969,778	77.79	98.95	96.53	47.15
M1-3	83,334,108	71,581,506	85.9	98.99	96.67	46.1
M2-1	68,487,140	63,008,552	92	98.77	95.95	45.76
M2-2	59,999,898	48,467,772	80.78	98.78	95.97	45.62
M2-3	83,400,560	69,073,336	82.82	98.91	96.42	46.2
M3-1	82,064,540	71,117,580	86.66	98.97	96.63	47.8
M3-2	77,068,190	65,893,684	85.5	98.91	96.4	47.45
M3-3	59,486,832	49,481,498	83.18	98.86	96.22	47.48
M4-1	58,993,002	39,712,432	67.32	99.05	96.74	46.5
M4-2	61,810,610	47,791,312	77.32	98.94	96.51	47.13
M4-3	81,157,528	66,044,302	81.38	98.96	96.57	46.37
M5-1	80,922,692	64,984,758	80.3	98.94	96.48	47.25
M5-2	68,679,784	56,712,078	82.57	98.96	96.58	47.62
M5-3	75,965,132	62,930,742	82.84	98.97	96.64	46.31
M6-1	85,321,660	71,734,596	84.08	98.9	96.37	45.89
M6-2	81,520,868	66,976,862	82.16	98.99	96.69	45.05
M6-3	72,073,174	52,279,938	72.54	99.04	96.8	47.8

Supplementary Table S4. Information of RNA-seq reads mapped with the reference transcriptome

Sample	Total Read pairs	Total mapped reads	Unique mapped reads	Multiple mapped reads
M1-1	32142809	27,847,857(86.64%)	6,604,630(20.55%)	21,243,227(66.09%)
M1-2	33484889	29,217,209(87.25%)	5,998,446(17.91%)	23,218,763(69.34%)
M1-3	35790753	31,284,138(87.41%)	8,271,507(23.11%)	23,012,631(64.30%)
M2-1	31504276	27,688,897(87.89%)	8,912,288(28.29%)	18,776,609(59.60%)
M2-2	24233886	21,140,768(87.24%)	6,693,518(27.62%)	14,447,250(59.62%)
M2-3	34536668	30,641,051(88.72%)	8,347,229(24.17%)	22,293,822(64.55%)
M3-1	35558790	31,917,431(89.76%)	5,043,997(14.18%)	26,873,434(75.57%)
M3-2	32946842	28,967,422(87.92%)	5,229,474(15.87%)	23,737,948(72.05%)
M3-3	24740749	21,650,151(87.51%)	3,362,738(13.59%)	18,287,413(73.92%)
M4-1	19856216	16,646,620(83.84%)	3,867,688(19.48%)	12,778,932(64.36%)
M4-2	23895656	20,724,055(86.73%)	3,899,188(16.32%)	16,824,867(70.41%)
M4-3	33022151	29,215,838(88.47%)	6,863,179(20.78%)	22,352,659(67.69%)
M5-1	32492379	28,608,292(88.05%)	4,590,724(14.13%)	24,017,568(73.92%)
M5-2	28356039	24,996,969(88.15%)	4,054,104(14.30%)	20,942,865(73.86%)
M5-3	31465371	26,801,555(85.18%)	6,902,129(21.94%)	19,899,426(63.24%)
M6-1	35867298	31,354,986(87.42%)	8,221,520(22.92%)	23,133,466(64.50%)
M6-2	33488431	29,044,274(86.73%)	9,002,281(26.88%)	20,041,993(59.85%)
M6-3	26139969	23,033,210(88.11%)	3,398,648(13.00%)	19,634,562(75.11%)

Supplementary Table S5. The significantly enriched pathways across different altitudes.

Comparison group	Pathway	Pathway ID	Gene number
M2-M1	Arginine biosynthesis	ko00220	36
	Nitrogen metabolism	ko00910	33
	Alanine, aspartate and glutamate metabolism	ko00250	44
	Protein processing in endoplasmic reticulum	ko04141	161
M4-M3	Glycolysis / Gluconeogenesis	ko00010	62
	Protein processing in endoplasmic reticulum	ko04141	83
M6-M5	Isoflavonoid biosynthesis	ko00943	9
	MAPK signaling pathway - plant	ko04016	31

Supplementary Table S6. The significantly enriched pathways at different developmental stages of seeds.

Comparison group	Pathway	Pathway ID	DEGs number
M3-M1	Glycolysis / Gluconeogenesis	ko00010	52
	Tyrosine metabolism	ko00350	18
	Fatty acid degradation	ko00071	23
	RNA degradation	ko03018	26
	Monoterpenoid biosynthesis	ko00902	7
	alpha-Linolenic acid metabolism	ko00592	18
	Pyruvate metabolism	ko00620	30
M5-M3	Isoflavonoid biosynthesis	ko00943	13
	Glutathione metabolism	ko00480	53
	MAPK signaling pathway - plant	ko04016	48
	Plant hormone signal transduction	ko04075	66
	Phenylpropanoid biosynthesis	ko00940	58
	Starch and sucrose metabolism	ko00500	78
	Biotin metabolism	ko00780	14
	Fatty acid biosynthesis	ko00061	32
	Monoterpenoid biosynthesis	ko00902	12
	Stilbenoid, diarylheptanoid and gingerol biosynthesis	ko00945	12
M5-M1	Glyoxylate and dicarboxylate metabolism	ko00630	41
	Cyanoamino acid metabolism	ko00460	37
	Flavonoid biosynthesis	ko00941	37
	Phenylpropanoid biosynthesis	ko00940	75
	Starch and sucrose metabolism	ko00500	100
	Fatty acid biosynthesis	ko00061	43
	Plant hormone signal transduction	ko04075	82
	Biotin metabolism	ko00780	17

MAPK signaling pathway - plant	ko04016	55
Glutathione metabolism	ko00480	58
Monoterpenoid biosynthesis	ko00902	15
Sesquiterpenoid and triterpenoid biosynthesis	ko00909	15
Thiamine metabolism	ko00730	19
Glyoxylate and dicarboxylate metabolism	ko00630	50
Cyanoamino acid metabolism	ko00460	46
Phenylalanine metabolism	ko00360	18
Isoflavonoid biosynthesis	ko00943	8
Stilbenoid, diarylheptanoid and gingerol biosynthesis	ko00945	12
Galactose metabolism	ko00052	28
Pyruvate metabolism	ko00620	73
Phenylalanine, tyrosine and tryptophan biosynthesis	ko00400	28
Terpenoid backbone biosynthesis	ko00900	29
DNA replication	ko03030	24

Supplementary Table S7. The DEGs in significantly enriched pathways at different altitude.

Genes	logFC M2-M1	logFC M4-M3	logFC M6-M5	Description	Pathway
m1_mix_transcript_24290	11.20	23.73	22.35	2,3-bisphosphoglycerate-independent phosphoglycerate mutase	Biosynthesis of amino acids, Biosynthesis of amino acids, Carbon metabolism, Glycolysis / Gluconeogenesis
m3_mix_transcript_8965	9.56	12.08	24.41	PREDICTED: 2,3-bisphosphoglycerate-independent phosphoglycerate mutase	Biosynthesis of amino acids, Biosynthesis of amino acids, Carbon metabolism, Glycolysis / Gluconeogenesis
m3_mix_transcript_22521	4.36	8.61	5.33	indole-3-glycerol phosphate synthase, chloroplastic-like isoform X1	Biosynthesis of amino acids
m4_mix_transcript_18991	23.81	11.28	11.11	fructose-bisphosphate aldolase 3, chloroplastic	Biosynthesis of amino acids, Carbon metabolism, Glycolysis / Gluconeogenesis, Carbon fixation in photosynthetic organisms
m5_mix_transcript_23721	9.69	10.01	13.16	s-adenosylmethionine synthase 1, partial	Biosynthesis of amino acids
m5_mix_transcript_857	3.74	5.11	6.89	TPA_asm: hypothetical protein HUJ06_012617	Biosynthesis of amino acids
m4_mix_transcript_19266	-2.89	-1.80	-2.85	Serine racemase	Biosynthesis of amino acids
m6_mix_transcript_19382	-1.69	-1.50	-1.86	hypothetical protein F0562_032151	Caffeine metabolism
m6_mix_transcript_17651	-5.50	-9.31	-8.00	PREDICTED: malate dehydrogenase, glyoxysomal	Carbon metabolism, Carbon fixation in photosynthetic organisms
m6_mix_transcript_28189	-27.10	-26.40	-9.50	malate dehydrogenase	Carbon metabolism, Carbon fixation in photosynthetic organisms

Supplementary Table S8. The DEGs in significantly enriched pathways at different developmental stages of seeds

Genes	logFC M3-M1	logFC M5-M3	logFC M5-M1	Description	Pathway
m1_mix_transcript_24314	1.95	2.46	4.42	hypothetical protein CISIN_1g044553mg	Vitamin B6 metabolism
m1_mix_transcript_9546	5.12	5.25	10.37	alpha-terpineol synthase	Monoterpenoid biosynthesis
m4_mix_transcript_19737	4.58	8.74	13.32	alpha-terpineol synthase	Monoterpenoid biosynthesis
m3_mix_transcript_21298	-6.53	-4.36	-10.92	endochitinase-like	MAPK signaling pathway - plant, Amino sugar and nucleotide sugar metabolism
m5_mix_transcript_20614	-5.11	-5.32	-10.44	endochitinase-like	MAPK signaling pathway - plant, Amino sugar and nucleotide sugar metabolism
m6_mix_transcript_11589	-5.39	-3.65	-9.04	endochitinase-like	MAPK signaling pathway - plant, Amino sugar and nucleotide sugar metabolism
m5_mix_transcript_23966	-4.91	-5.36	-10.29	LOW QUALITY PROTEIN: endochitinase A2-like	MAPK signaling pathway - plant, Amino sugar and nucleotide sugar metabolism
m5_mix_transcript_13304	-6.38	-5.92	-12.29	hypothetical protein PHAVU_003G147600g	Plant hormone signal transduction
m5_mix_transcript_21162	-1.71	-1.75	-3.46	hypothetical protein F0562_035510	MAPK signaling pathway - plant, Plant hormone signal transduction
m5_mix_transcript_7357	-1.62	-2.00	-3.61	hypothetical protein F0562_016784	Plant hormone signal transduction
m6_mix_transcript_14068	-6.91	-5.52	-12.44	hypothetical protein B456_004G030700	Plant hormone signal transduction
m6_mix_transcript_18648	-6.93	-4.33	-11.26	hypothetical protein B456_004G030700	Plant hormone signal transduction
m6_mix_transcript_30853	-8.30	-6.31	-14.62	PREDICTED: probable xyloglucan endotransglucosylase/hydrolase protein 23	Plant hormone signal transduction

Supplementary Table S9. Statistical chart of lipid metabolism pathway

Pathway	Pathway ID	Gene number		
		M3-M1(up/down)	M5-M3(up/down)	M5-M1(up/down)
Fatty acid biosynthesis	ko00061	10(6/4)	32(29/3)	43(37/6)
Fatty acid elongation	ko00062	2(1/1)	1(1/0)	7(7/0)
Fatty acid degradation	ko00071	23(16/7)	22(12/10)	28(18/10)
Cutin, suberine and wax biosynthesis	ko00073	2(1/1)	3(0/3)	5(2/3)
Steroid biosynthesis	ko00100	4(2/2)	15(7/8)	16(9/7)
Glycerolipid metabolism	ko00561	7(3/4)	15(6/9)	13(8/5)
Glycerophospholipid metabolism	ko00564	9(6/3)	24(18/6)	28(23/5)
Ether lipid metabolism	ko00565	0(0/0)	6(4/2)	8(8/0)
Arachidonic acid metabolism	ko00590	2(0/2)	7(3/4)	4(1/3)
Linoleic acid metabolism	ko00591	3(2/1)	7(3/4)	7(5/2)
alpha-Linolenic acid metabolism	ko00592	18(12/6)	26(13/13)	24(17/7)
Sphingolipid metabolism	ko00600	3(0/3)	6(3/3)	4(1/3)
Biosynthesis of unsaturated fatty acids	ko01040	6(3/3)	6(2/4)	8(6/2)

Supplementary Table S10. Key genes associated with fatty acid biosynthesis

DEGs	Mean FPKM			logFC			Gene	Description	KO ID
	M1	M3	M5	M3-M1	M5-M3	M5-M1			
m2_mix_transcript_10940	29.31	19.89	1.19	0.07	3.96	4.03	BC	Biotin carboxylase 1, chloroplastic isoform 1	K01961
m3_mix_transcript_4548	46.46	33.30	8.57	0.02	1.95	1.98		biotin carboxylase 2, chloroplastic	
m3_mix_transcript_11452	7.95	16.92	0.10	-1.76	7.43	5.68		acetyl-CoA carboxylase BC subunit	
m1_mix_transcript_22034	35.65	9.05	5.50	1.48	0.76	2.24		acetyl-CoA carboxylase BC subunit	
m2_mix_transcript_22121	13.47	11.98	1.10	-0.35	3.30	2.94	BCCP	biotin carboxyl carrier protein of acetyl-CoA carboxylase, chloroplastic-like	K02160
m4_mix_transcript_18819	50.49	51.19	5.86	-0.49	2.95	2.45		Biotin_lipoyl domain-containing protein	
m2_mix_transcript_10032	3.32	0.71	3.54	1.80	-2.16	-0.35	α-CT	acetyl-coenzyme A carboxylase carboxyl transferase subunit alpha, chloroplastic-like	K01962
m4_mix_transcript_1363	16.62	8.52	3.53	0.46	1.22	1.68		acetyl-coenzyme A carboxylase carboxyl transferase subunit alpha, chloroplastic	
m4_mix_transcript_12209	28.09	18.06	3.73	0.14	2.19	2.33	MACT	malonyl-CoA:ACP transacylase	K00645
m4_mix_transcript_22728	37.31	25.20	4.77	0.03	2.37	2.41		malonyl-CoA-acyl carrier protein transacylase, mitochondrial isoform X2	
m5_mix_transcript_8515	101.70	108.94	14.94	-0.58	2.71	2.12	KAS I	3-oxoacyl-[acyl-carrier-protein] synthase I, chloroplastic	K09458
m1_mix_transcript_11058	48.21	13.87	0.63	1.30	4.29	5.59		3-oxoacyl-[acyl-carrier-protein] synthase I, chloroplastic	
m3_mix_transcript_	43.3	52.6	2.5	-0.81	4.30	3.50		3-oxoacyl-[acyl-carrier-protein] synthase I, chloroplastic-like	

18104	5	3	8						
m2_mix_transcript_7466	42.88	53.69	9.60	-0.80	2.38	1.58		3-oxoacyl-[acyl-carrier-protein] synthase II, chloroplastic-like	
m2_mix_transcript_7277	12.95	13.75	1.74	-0.57	2.76	2.18	KAS II	3-oxoacyl-[acyl-carrier-protein] synthase II, chloroplastic-like	
m2_mix_transcript_15999	19.64	0.18	2.71	6.36	-4.10	2.25		ketoacyl-ACP synthase II	
m2_mix_transcript_29102	0.00	18.01	0.34	-11.96	5.51	-6.47		3-oxoacyl-[acyl-carrier-protein] reductase 4-like	
m5_mix_transcript_10844	114.88	87.77	23.05	-0.13	1.92	1.79	KRA	3-oxoacyl-[acyl-carrier-protein] reductase 4-like	K00059
m1_mix_transcript_26653	2.55	0.76	0.00	1.15	6.24	7.40		3-oxoacyl-[acyl-carrier-protein] reductase 4-like	
m1_mix_transcript_28514	8.52	8.20	0.25	-0.52	4.95	4.42	HAD	3-hydroxyacyl-[acyl-carrier-protein] dehydratase FabZ-like	K02372
m2_mix_transcript_32498	11.59	8.64	1.19	-0.08	2.71	2.62		3-hydroxyacyl-[acyl-carrier-protein] dehydratase FabZ-like	
m4_mix_transcript_22439	46.69	64.72	0.83	-0.99	6.12	5.11	EAR	Enoyl-[acyl-carrier-protein] reductase [NADH], chloroplastic	K00208
m4_mix_transcript_2041	16.12	12.21	29.95	-0.12	-1.34	-1.45		long chain acyl-CoA synthetase 6, peroxisomal-like	
m6_mix_transcript_4651	12.90	7.82	3.07	0.27	1.28	1.54		long chain acyl-CoA synthetase 4	
m2_mix_transcript_22163	7.06	2.54	0.64	0.89	1.91	2.81	LACS	long chain acyl-CoA synthetase 2 isoform X2	K01897
m6_mix_transcript_4395	34.81	18.61	52.23	0.41	-1.50	-1.09		Long chain acyl-CoA synthetase	
m1_mix_transcript_25700	14.46	8.92	2.69	0.15	1.63	1.78		long chain acyl-CoA synthetase 9, chloroplastic	
m1_mix_transcript_	6.21	1.43	0.3	1.52	2.12	3.65		long chain acyl-CoA synthetase 2	

5353			1						
m3_mix_transcript_ 11271	0.04	7.33	0.4 9	-8.64	3.88	-4.74	SAD	stearoyl-ACP desaturase	K039 21
m3_mix_transcript_ 6098	0.09	1.21	0.3 5	-4.47	1.54	-2.95		stearoyl-ACP desaturase	
m4_mix_transcript_ 28443	0.00	0.00	21. 67	0.00	-23.7 5	-24.3 2		stearoyl-ACP desaturase	
m1_mix_transcript_ 365	6.56	3.42	0.1 7	0.40	4.16	4.57		stearoyl-ACP desaturase	
m3_mix_transcript_ 9516	24.9 1	56.0 0	21. 77	-1.62	1.30	-0.32		plastid acyl-ACP thioesterase	K107 81
m2_mix_transcript_ 21194	108. 63	71.0 4	12. 71	0.16	2.29	2.44		oleoyl-acyl carrier protein thioesterase 1, chloroplastic-like	K107 82
