# Supplementary figures and images for: Integrative Analysis of Iso-Seq and RNA-Seq Identifies Key Genes Related to Fatty Acid Biosynthesis and High-Altitude Stress Adaptation in Paeonia delavayi
Source: Genes (Basel). 2025 Jul 30;16(8):919. doi: 10.3390/genes16080919 (PMC12385284; doi:10.3390/genes16080919)

A

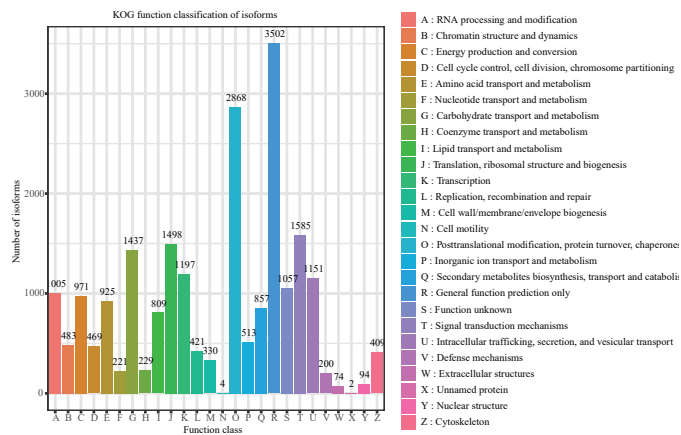

B

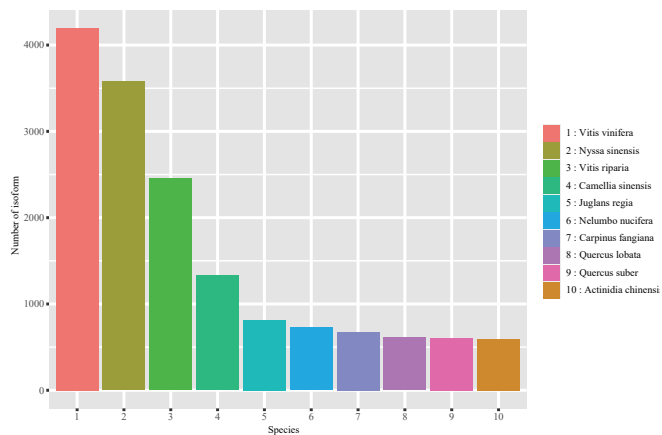

C

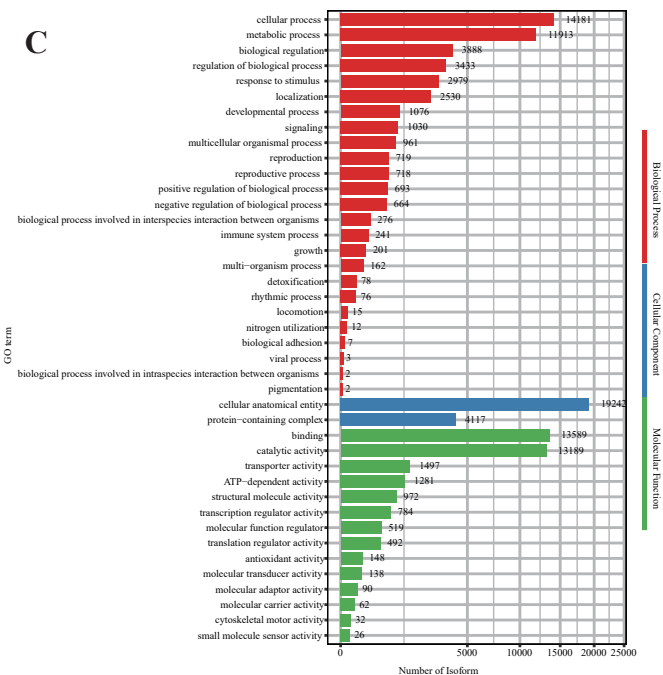

D

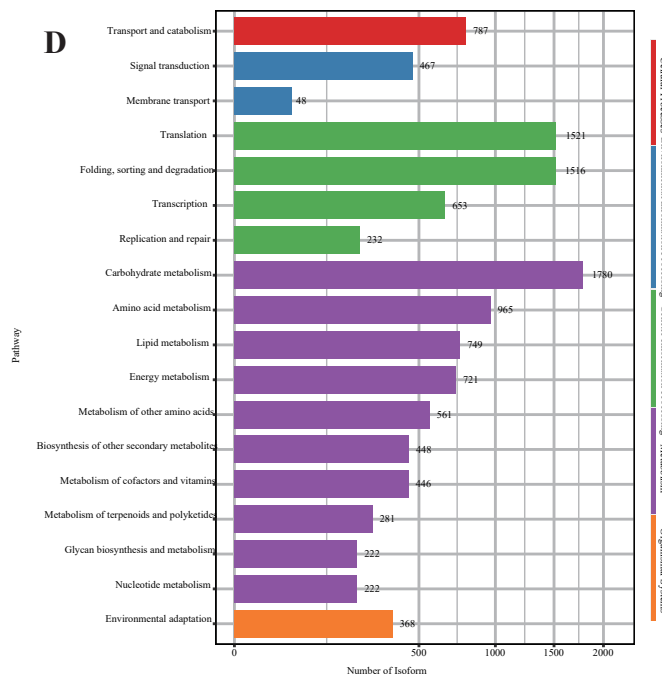

Supplement: Supplementary file 1 [file genes-16-00919-s001.zip › Supplementary files/Supplementary figure/Figure S1.pdf]

A

M2-vs-M1

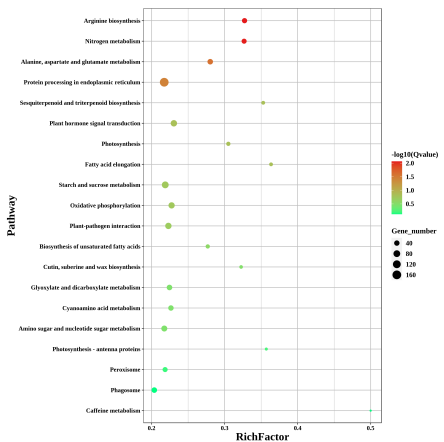

M4-vs-M3

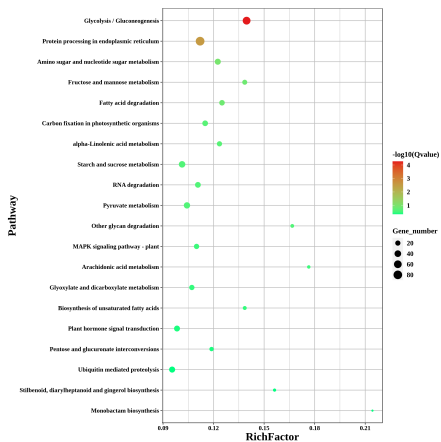

M6-vs-M5

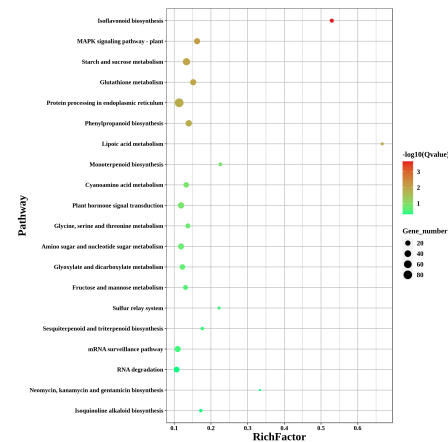

B

M3-vs-M1

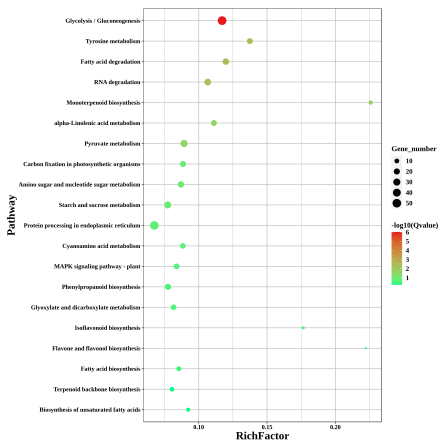

M5-vs-M3

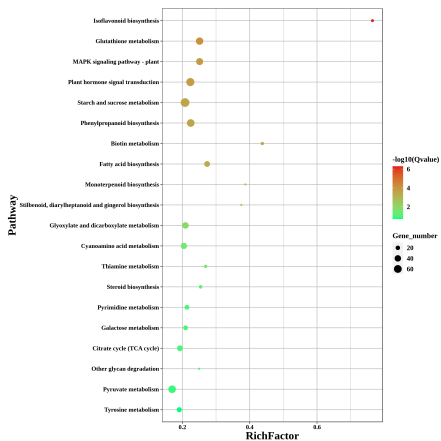

M5-vs-M1

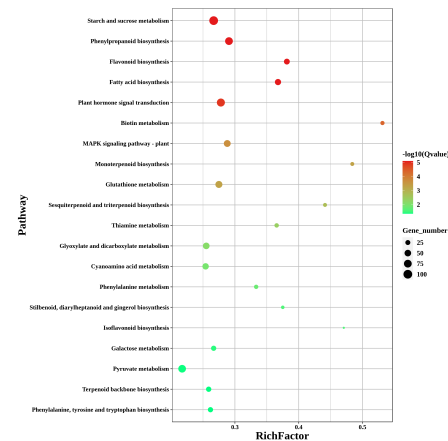

Supplement: Supplementary file 1 [file genes-16-00919-s001.zip › Supplementary files/Supplementary figure/Figure S2.pdf]
